# Anti-biofilm studies of synthetic imidazolium salts on dental biofilm in vitro

**DOI:** 10.1080/20002297.2022.2075309

**Published:** 2022-05-17

**Authors:** Ting Pan, Feng-Shou Liu, Huancai Lin, Yan Zhou

**Affiliations:** a Hospital of Stomatology, Guanghua School of Stomatology, Sun Yat-sen University, Guangzhou, China; b Guangdong Key Laboratory for Dental Disease Prevention and Control, Sun Yat-sen University, Guangzhou, China; c School of Chemistry and Chemical Engineering, Guangdong Pharmaceutical University, Zhongshan, China

**Keywords:** Dental caries, antimicrobials, antibiofilm, imidazolium salts

## Abstract

**Objective:**

Biofilm formation under cariogenic conditions contributes to dental caries development, in which *Streptococcus mutans* (*S. mutans*) is regarded as the major cariogenic bacteria. Here, we synthesized a series of imidazolium salts. Their properties of antimicrobial and anti-biofilm were investigated.

**Methods:**

The microdilution method crystal violet staining, and cell counting Kit-8 assay were used to screen imidazolium salts. Then, the bacterial composition in multi-species biofilm composed of *S. mutans, Actinomyces naeslundii,* and *Streptococcus gordonii* was quantified by quantitative PCR. The exopolysaccharide and morphology of the structure of multi-species biofilm were further observed by confocal laser scanning microscopy and scanning electron microscope, respectively.

**Results:**

Imidazolium salts exhibited highly antimicrobial activity against oral pathogens, especially for *S. mutans* . Compounds with *ortho*-diisopropyl and *para*-methoxyl on N-moieties as well as bearing ancenaphthyl skeleton (C5) showed the lowest cytotoxicity and most efficient anti-biofilm activity. C5 inhibited approximately 50% of multi-species biofilm at 0.98 μg/mL. Notably, C5 resulted in 98.97% live *S. mutans* and 77.65% *A. naeslundii* decreased. Furthermore, the exopolysaccharide was reduced by 88%, along with a sparse and scattered microstructure.

**Conclusion:**

The imidazolium salts present low cytotoxicity and remarkable antimicrobial activity against *S. mutans* in multi-species biofilm, suggesting that they may have a great potential in anti-biofilm clinical applications.

## Introduction

Biofilm refers to microorganisms living in complex three-dimensional structures composed of cells, polysaccharides, and other components such as proteins, extracellular DNA, and lipids [1]. Biofilm confers adaptive resistance and physical protection to microorganisms, and plays a vital role in pathogenicity and drug resistance [2,3]. It is reported that 80% of chronic infections are related to biofilms [4]. Therefore, it is an important issue to explore and develop effective strategies to control biofilm. Physical-mechanical removal as well as chemotherapy are recognized to impede biofilm formation or eradicate biofilms [5–7].

**Scheme 1.**
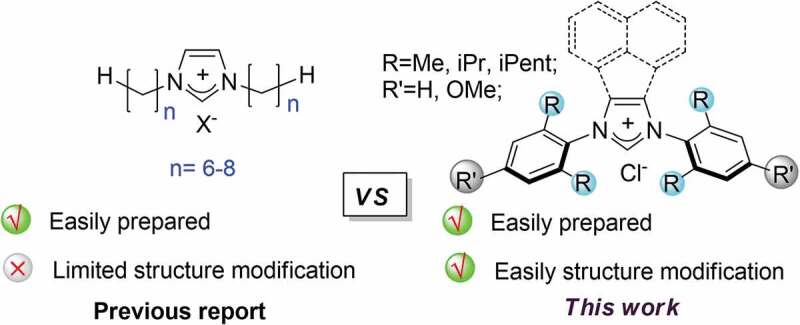
The design of the imidazolium salts.

Among the antimicrobial strategies of chemotherapy, small-molecule drugs are identified to be efficient tools to prevent biofilm formation due to relatively low cost and ease of control and use [8]. Therefore, a plethora of small-molecule compounds, such as phenols, imidazoles, furanone, and indole, were described [9]. In this context, the imidazole derivatives, such as 2-aminoimidazole compounds, have been extensively investigated as scaffolds for synthetic anti-biofilm inhibitors [9–11]. It is noteworthy that imidazolium saltswith alkyl side chains, have been recognized as valuable candidates for their flexible structural modification. In the past decades, it is highlighted that these compounds exhibited broad-spectrum antimicrobial activities, including *Staphylococcus aureus, Escherichia coli, Salmonella typhimurium, and Candida albicans*, along with low minimum inhibitory concentration (MIC) values [12]. Nevertheless, considerable cytotoxicity was frequently inevitable due to the limited structure modification of the cationic structure (Scheme 1). As far as concerned, few examples have been successfully achieved in the balance of the cytotoxicity and antimicrobial activity to date.

On the other hand, dental caries and periodontal disease result from a microecology dysbiosis in the dental biofilm [13,14]. Dental caries continues to be the most prevalent condition, especially untreated dental caries in permanent teeth has affected 2.5 billion people globally [15]. The progress of caries starts with some early colonizers that can rapidly adhere to the tooth surface, and then co-adhere with other microorganisms, which represents a microbial ecosystem [16]. *Streptococcus mutans* (*S. mutans*), widely detected in dental biofilm, is well-recognized as one of the major cariogenic bacteria [17,18]. The ability to synthesize exopolysaccharides, the main constituents in the matrix of cariogenic dental biofilm, promotes the formation of a three-dimensional scaffold and enhances mechanical stability [19]. Its strong acidogenicity and acid-resistivity work coordinately to alter biofilm microecology [20]. Besides, much research also has identified non-mutans streptococci and *Actinomyces* are closely involved with the initiation of caries [21]. Once a biofilm is established, the microorganisms embedded and sheltered in the matrix are recalcitrant to antimicrobials, making it difficult to disrupt or eradicate [22]. Recently, a type of imidazolium carbene copper compounds has been succeeded developed by us, which demonstrated a greatly potential of broad-spectrum inhibitors toward oral bacteria [23]. Nevertheless, the inherent of toxicity of copper ion impedes their further application. Keeping this in mind, we envisioned that a rational structure design on N-moieties and backbones of the imidazolium salts would provide excellent inhibitory effects and diminish the cytotoxicity at the same time (Scheme 1).

Herein, we presented imidazolium salts with several different side chain structures and their properties against oral microorganisms. To the best of our knowledge, it is the first report on the potential application of imidazolium salts in the control of dental mono-species and multi-species biofilm.

## Materials and methods

The imidazolium salts of C1-C4 were synthesized according to the previously reported procedures [24].

### The procedure for the synthesis of imidazolium salt of C5

Under nitrogen atmosphere, a mixture of *α*-diimine compound (1.00 mmol) and chloromethyl ethyl ether (4 mL) was stirred at 100°C overnight. Then, the solution was cooled to room temperature, treated with Et_2_O and stirred for another 1 h, resulting the formation of yellowish precipitate. The solid was isolated by filtration and washed with anhydrous Et_2_O three times. The desired imidazolium salt of C5 was obtained in 82% yield. ^1^H NMR (400 MHz, CDCl_3_) δ 11.21 (br, 1 H), 8.04 (d, *J* = 8.2 Hz, 2H), 7.61 (t, *J* = 7.6 Hz, 2H), 7.31 (d, *J* = 7.9 Hz, 2 H), 6.94 (s, 4 H), 3.96 (s, 6 H), 2.71–2.63 (m, 4 H), 1.35 (d, *J* = 6.5 Hz, 12 H), 1.16 (d, *J* = 6.5 Hz, 12 H). ^13^C NMR (101 MHz, CDCl_3_) δ 161.9 146.5, 137.7, 134.4, 130.5, 130.4, 129.9, 128.2, 123.1, 122.81, 121.8, 110.2, 55.5, 29.5, 24.6, 23.4.

### Microbial strains and incubation conditions

*S. mutans* UA159, *Streptococcus gordonii* ATCC10558 (*S. gordonii), Lactobacillus acidophilus* ATCC4356 (*L. acidophilus), Lactobacillus casei* ATCC393 (*L. casei), Actinomyces naeslundii* DSM17233 (*A.naeslundii), Enterococcus faecalis* OG1RF (*E. faecalis), Candida albicans* SC5314 (*C. albicans), Aggregatibacter actinomycetemcomitans* ATCC43717 (*A.a), Fusobacterium nucleatum* ATCC10953 (*F. nucleatum*) were used as representative oral pathogens to test antimicrobial effects of imidazolium salts. *F. nucleatum* grown under completely anaerobic condition in brain heart infusion (BHI, BD, USA) solution supplemented with 0.5% yeast extract (Oxoid, UK), 0.04% L-cysteine (Sigma, USA), 5 μg/mL hemin (Macklin, CHN) and 1 μg/mL vitamin K1 (Aladdin, CHN), while *C. albicans* grown in sabouraud’s dextrose broth (SDB, HKM, CHN) and were incubated aerobically with shaking at 200 rpm. The other strains were cultured anaerobically (90% N_2_, 5% H_2_ and 5% CO_2_) at 37°C in BHI to reach the mid-logarithmic phase.

### Antimicrobial of imidazolium salts in planktonic microorganisms

The minimum inhibitory concentration (MIC) of imidazolium salts against planktonic pathogens was evaluated by broth microdilution method [25]. In brief, all bacteria except fungus were diluted to 5 × 10^5^ CFU/mL, while *C. albicans* diluted to 5 × 10^3^ CFU/mL. The 96-well microtiter plates contained imidazolium salts with two-fold serial dilutions from 500 μg/mL to 0.24 μg/mL and microorganisms were incubated for 24 h at 37°C. Non-treated bacteria with broth were set as the negative control, and chlorhexidine (CHX) as the positive control. Microbial growth was measured by naked eye.The MIC is defined as the lowest concentration of the antimicrobial agent that prevents visible growth of a microorganism.

### Cytotoxicity assays of imidazolium salts

Cytotoxicity of imidazolium salts was determined using the Cell Counting Kit-8 (CCK-8, Dojindo, Japan). Human gingival epithelial cells (HGECs) grown in defined keratinocyte serum-free medium (Gibco, USA) were pre-cultured in a 96-well flat-bottom plate (10000 cells/well). After 24 h incubating in a 5% CO_2_ atmosphere at 37°C, the HGECs were washed with sterile PBS. Medium mixed with imidazolium salts was added to each well and incubated for another 24 h. The blank or negative group was only replaced with a fresh medium. Then, cells were washed with sterile PBS and substituted with 10 μL of CCK-8 reagent diluted in 100 μL of cell culture medium. After another 2 h incubation, the OD_450_ absorbance values were performed. The cell viability was calculated as cell viability% = (OD test-OD blank)/(OD negative – OD blank) × 100%.

### Anti-biofilm of imidazolium salts in multi-species biofilm

#### Minimum biofilm inhibitory concentration (MBIC) of imidazolium salts

First, the imidazolium salts were tested in mono-species biofilm of *S. mutans*. Then, the imidazolium salts with the highest antimicrobial effectiveness and lowest cytotoxicity were selected in multi-species biofilm model. In general, MBIC of imidazolium salts was measured by crystal violet (CV) assays. Bacteria in BHI with 1% sucrose (BHIS) were adjusted to 2 × 10^7^ CFU/mL to form mono-species biofilm, and the biofilm susceptibility of each strain in multi-species biofilm model was assessed.

Next, a mixture of three strains of bacteria were prepared to form multi-species biofilm. The bacteria included *S. mutans, S. gordonii,* and *A. naeslundii*. The multi-species biofilm model was built by adding each species with 1:1:1 volume in BHIS at the same time. The final bacterial concentration of each bacterium was also adjusted to 2 × 10^7^ CFU/mL. A 200-μL bacterial suspension supplemented with imidazolium salts or CHX was added in plates and cultured anaerobically at 37°C. After 24 h incubation, the supernatant was discarded and the adherent biofilm was washed by sterile PBS, followed by fixed with 100% methyl alcohol for 15 min. The biofilm was then stained using 0.1% CV (Sigma, USA) for another 15 min. Excess dye was removed and washed by PBS until the blank well appeared colorless. Finally, 200 μL 95% ethanol was added to each well to dissolve the dye. After 20 min, the absorbance at OD_595_ was recorded. MBIC_90_ is defined as the lowest concentration to inhibit 90% biofilm, while MBIC_50_ is defined to inhibit 50% biofilm.

#### Exopolysaccharide(EPS) assay in multi-species biofilm

EPS in multi-species biofilm after being treated with imidazolium salt was assayed by using confocal laser scanning microscopy (CLSM). Multi-species biofilm formed as described previously. Briefly, a mixed suspension of three bacteria was added to the confocal dish with a final bacterial concentration of 2 × 10^7^ CFU/mL. Imidazolium salts or CHX was also added at the meantime and cultured anaerobically at 37°C for 24 h. For EPS staining, 2.5 μM Alexa Fluor 647 (Invitrogen, USA) labelled dextran conjugate was added to the bacterial solution at the beginning, and subsequent processes were protected from the light. After 24 h of biofilm formation, the supernatant was discarded and replaced with PBS containing 2.5 μM SYTO9 (Invitrogen, USA). Then, the excess dye was removed using PBS after 15 min incubation. A CLSM equipped with a 20× lens (Olympus, JPN) was utilized for capturing fluorescence images. The quantification of EPS/bacteria volume was performed with COMSTAT2, a plugin to Image J [26,27].

#### Compositional analysis of total/live bacteria in multi-species biofilm

Total and live bacteria in multi-species biofilm were enumerated using a live-dead PCR method, a technique that has been shown to differentiate viable and dead cells [28–30]. Propidium monoazide (PMA), a light-reactive dye, can only penetrate to dead cells and bind covalently with DNA [30]. Based on this covalent bonding, amplification of DNA in dead cells can be prevented in PCR, and only live cells can be detected.

In brief, multi-species biofilm samples were formed as described above. 20 mM PMA (Biotium, USA) was added in samples, while no PMA controls were also included to assess total bacteria. After 10 min incubation in darkness, all samples were exposed to a 500 W halogen light for 15 min. Ultrasonic vibration (40 kHz, 2 min) was used to induce biofilm dispersion. Bacterial DNA was extracted from the ultrasonic-dispersed biofilm using the QIAamp Bacteria DNA mini kit (TIANGEN, CN). The purity and concentration of extracted DNA were determined by NanoDrop spectrophotometer (Thermo, USA). The concentration of total DNA extracted is 30–100 ng/μL. For qPCR, 2 μL DNA was added in 10 μL SYBR ®Premix Ex Taq^TM^ II (2×) (Takara, Japan), 6.4 μL RNase-free water, and 0.8 μL of 100 μM forward/reverse primers for each bacterial species. The sequences of primers used for qPCR were presented in Table 1. Amplification and quantification of extracted DNA were performed in a Light Cycler 96 system (Roche, CH) with the following cycle conditions: 95°C for 1 min, followed by 40 cycles of 95°C for 5 s, 60°C for 20 s, 70°C for 1 s and a final melting of 95°C for 15 s and 60°C for 1 min. For the standard curve, we made 10-fold dilutions of each bacterium after incubation and counted them on agar plates. Meanwhile, the DNA of diluted bacteria was extracted for qPCR. A standard curve was established utilizing the Ct values of a single bacterial strain and its corresponding CFU values (Figure S1 in Supplementary material).

**Table 1.** Sequence of primers used in this study

Bacteria	Primer sequence (5’-3’)	Size	Tm	Concentration
*S. mutans*	F:GCCTACAGCTCAGAGATGCTATTCT [49] R:GCCATACACCACTCATGAATTGA	114 bp	62 64	0.4 μM 0.4 μM
*S. gordonii*	F:CGGATGATGCTAATCAAGTGACC [50] R:GTTAGCTGTTGGATTGGTTGCC	176 bp	58 58	0.4 μM 0.4 μM
*A. naeslundii*	F:CCTGGGAAAGATTGCGCCTT R: ACCAACAAGCTGATAGGCCG	70 bp	57 60	0.4 μM 0.4 μM

#### Biofilm structure assay by using scanning electron microscope (SEM)

The multi-species biofilm was fixed with 2.5% glutaraldehyde for 3 h and then washed with sterile PBS and dehydrated by an alcohol gradient (30%, 50%, 70%, 90%, and 100%). Samples were dried by lyophilization and metal spraying following treated with tert‐butanol. The samples were observed at 2,000× and 20,000× magnification by SEM (FEI, CZ).

### Statistic analysis

All experiments were independently repeated in triplicate. GraphPad Prism version 7.0 and SPSS 25.0 were utilized to analyze the data. One-way ANOVA was performed on multiple sets of samples to determine significant differences, followed by a Turkey’s test. *P* < 0.05 was considered as statistical significance.

## Results

### Chemical structure of synthetic imidazolium salts

**Figure 1.**
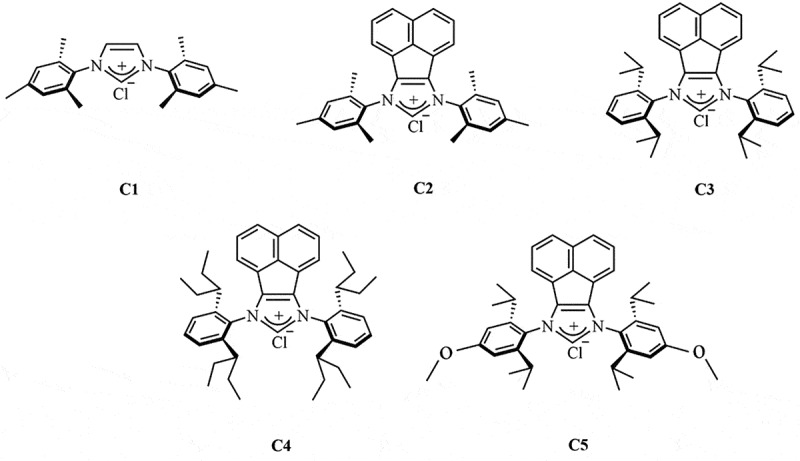
Molecular structure of imidazolium salts (C1-C5).

To reveal the impact of the chemical modification on the properties of the antimicrobial activity, a series of imidazolium salts were synthesized. As shown in Figure 1, the structural difference between C1 and C2 was the change in backbone, wherein the 1,2-dihyhrogen backbone for C1, while acenaphthyl for C2 instead. Moreover, different kinds of *ortho*-alkyl substituents were installed on the *N*-aryl moieties for C3 and C4, while *para*-methoxyl-functionalized C5 was further fabricated for comparison.

### Inhibitory effects of imidazolium salts on planktonic microorganisms

With these imidazolium salts in hand, the antimicrobial activity of these compounds was then screened. As shown in Table 2, it was shown that the MIC of C1 complex exerted in a significantly high level with the values of 125–500 μg/mL. In contrast, the other four complexes of C2-C5 exhibited great inhibitory properties against oral pathogens, with MIC values of 0.24–31.25 μg/mL. Moreover, C3 and its analogues of C4-C5 with various substitutions on the N-aryl moiety were found to have lower inhibitory concentrations against all tested bacteria except *F. nucleatum*. It is noteworthy that among these compounds investigated, C5 provided superior antibacterial activity against *S. mutans* to C3 and C4. Furthermore, C3-C5 performed equivalent to or even better than CHX in antimicrobial potency, especially against *C. albicans, L. casei, L. acidophilus, E. faecalis, A. a*. Based on the satisfying properties in antimicrobial potency, C2-C5 were selected for further evaluation on anti-biofilm activity and cytotoxicity.

**Table 2.** MIC values of C1- C5 on oral pathogens (μg/mL)

Bacteria	C1	C2	C3	C4	C5	CHX
*S. mutans*	125	0.49	0.98	1.95	0.49	0.49
*S. gordonii*	250	3.91	1.95	0.98	0.98	1.95
*C. albicans*	500	3.91	1.95	0.98	1.95	7.81
*L. casei*	125	31.25	0.49	0.49	0.49	31.25
*L. acidophilus*	500	31.25	0.98	0.49	0.49	15.63
*E. faecalis*	500	7.81	3.91	3.91	1.95	7.81
*A. naeslundii*	500	31.25	0.98	0.24	0.49	0.98
*A. a*	125	3.91	0.49	0.98	0.98	3.91
*F. nucleatum*	250	31.25	15.63	NS*	15.63	7.81

* = no significance

### Inhibition of *S.mutans* biofilm and cytotoxicity on HGECs

The suppression of *S. mutans* biofilm formation was assessed by CV assay, and MBIC values were quantified. The MBIC_90_ of C2 and C5 appeared the same anti-biofilm activity as CHX, which was 0.98 μg/mL. Nevertheless, C3 and C4 complexes inhibited *S. mutans* biofilm with relatively higher MBIC_90_ values, which were 1.46 μg/mL and 1.95 μg/mL, respectively.

**Figure 2.**
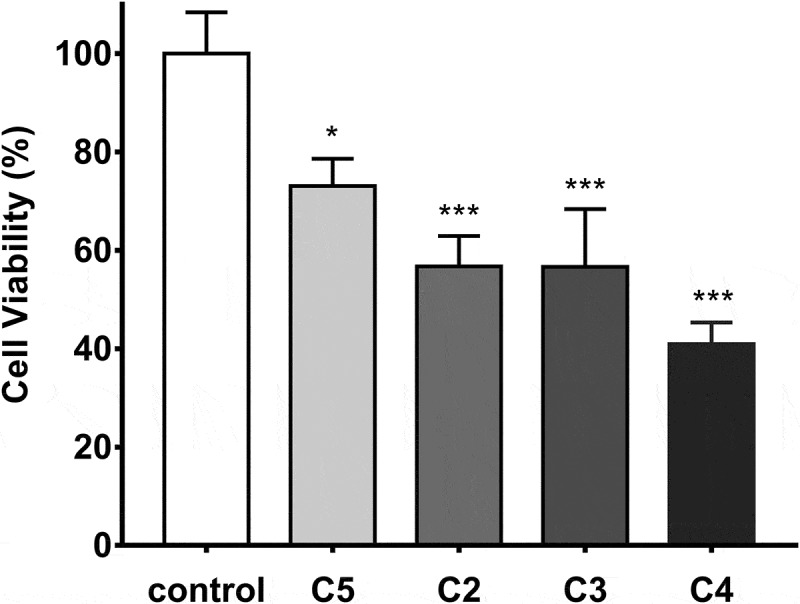
Cytotoxicity of imidazolium salts on HGECs. Cell viability was performed by CCK-8 after being treated with C2, C3, C4, and C5 for 24 h. Data are represented as means ± standard deviations. *, *P* < 0.05; ***, *P* < 0.001.

The cytotoxicity of each imidazolium salts on HGECs was further confirmed under its MBIC_90_. In Figure 2, it was found that C5 presented low cytotoxicity toward HGECs, with 73.07% cell viability after 24 h incubation (*P* < 0.05). The other imidazolium salts C2, C3, and C4 presented relatively higher cytotoxicity to HCECs, with only 56.71%, 56.64%, and 41.92% cell viability retained, respectively. From our assessment of antimicrobial tests and cytotoxicity results, C5 was demonstrated better biological properties in these synthetic imidazolium salts. Therefore, C5 was screened to determine the ability of inhibiting multi-species biofilm in subsequent experiments.

### Anti-biofilm effects of imidazolium salts against multi-species biofilm

In mono-species biofilm, C5 and CHX presented equipotent anti-biofilm effects against *S. mutans* and *A. naeslundii*, resulting in 90% biofilm inhibition at 0.98 μg/mL (Table 3). However, the MBIC_90_ for *S. gordonii* of these two agents was slightly different, with 1.47 μg/mL of C5 and 2.45 μg/mL of CHX. Moreover, in multi-species biofilm, the MBIC_50_ of C5 was 0.98 μg/mL, while CHX was 1.95 μg/mL.

**Table 3.** MBIC of C5 and CHX against *S. mutans, A. naeslundii, and S. gordonii*, mono-/multi-species biofilm

	MBIC_90_(μg/mL)			MBIC_50_(μg/mL)
	*S. mutans*	*A. naeslundii*	*S. gordonii*	Mixed
C5	0.98	0.98	1.47	0.98
CHX	0.98	0.98	2.45	1.95

### Total/live bacterial composition in multi-species biofilm

**Figure 3.**
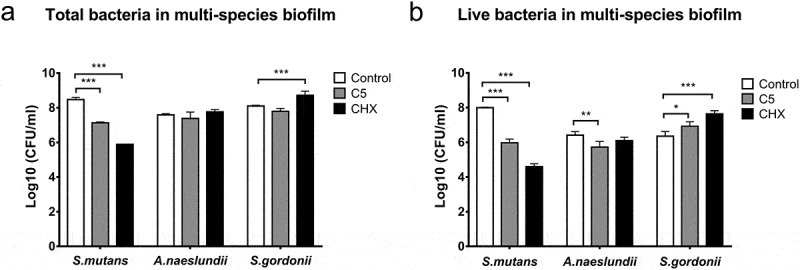
Effects of C5 and CHX on the amount of bacteria in multi-species biofilm. (a-b) total/live bacteria in multi-species biofilm of *S. mutans, S. gordonii*, and *A. naeslundii*. Data are represented as means ± standard deviations. *, *P* < 0.05; **, *P* < 0.01; ***, *P* < 0.001.

The effects of C5 and CHX on the microbial composition of multi-species biofilm were further investigated (Figure 3).

For *S. mutans*, the total bacteria were 3.02 × 10^8^ CFU/mL in control group, which were decreased by 95.52% in C5 group (*P* < 0.001) and 99.74% in CHX group (*P* < 0.001). For *A. naeslundii*, the total bacteria were not decreased in both treated groups. For *S. gordonii*, the total bacteria showed a 4.58-fold increase in CHX group (*P* < 0.001) compared with the control (1.26 × 10^8^ CFU/mL).

Moreover, the viable *S. mutans* bacteria were 9.94 × 10^7^ CFU/mL in control group, which were decreased by 98.97% in C5 group (*P* < 0.001) and 99.96% in CHX group (*P* < 0.001).

However, the viable *A. naeslundii* bacteria was decreased by 77.65% in C5 group (*P* < 0.05) compared with the control (2.78 × 10^6^ CFU/mL). Surprisingly, the viable *S. gordonii* bacteria in C5 and CHX group achieved a 3.71-fold (*P* < 0.05) and 17.83-fold (*P* < 0.001) increase, respectively.

Conclusively, C5 and CHX had excellent inhibitory effects against *S. mutans* in the treated biofilm, and the inhibition percentage could reach more than 98%. Besides, C5 inhibited live bacteria of *A. naeslundii* but CHX could not inhibit it.

### The change in EPS in multi-species biofilm

**Figure 4.**
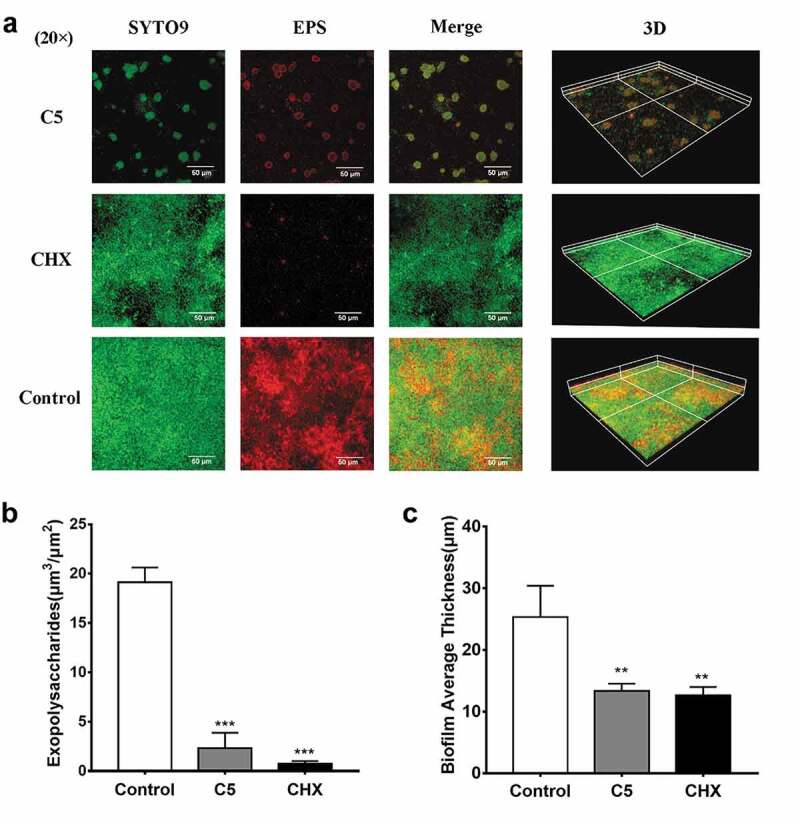
The CLSM images of treated multi-species biofilm. (a) The green indicated the microorganism; the red indicated the EPS. (b-c) The EPS and biofilm average thickness were quantified. Data are represented as means ± standard deviations.**, *P* < 0.01; ***, *P* < 0.001.

C5 and CHX both reduced glucans production by *S. mutans* (Figure 4). C5 leads an 88% (*P* < 0.001) decrease of EPS, while CHX resulted in 96% (*P* < 0.001). Biofilm average thickness was declined to 47% (*P* < 0.05) after C5 treatment and 50% (*P* < 0.05) after CHX treatment.

**Figure 5.**
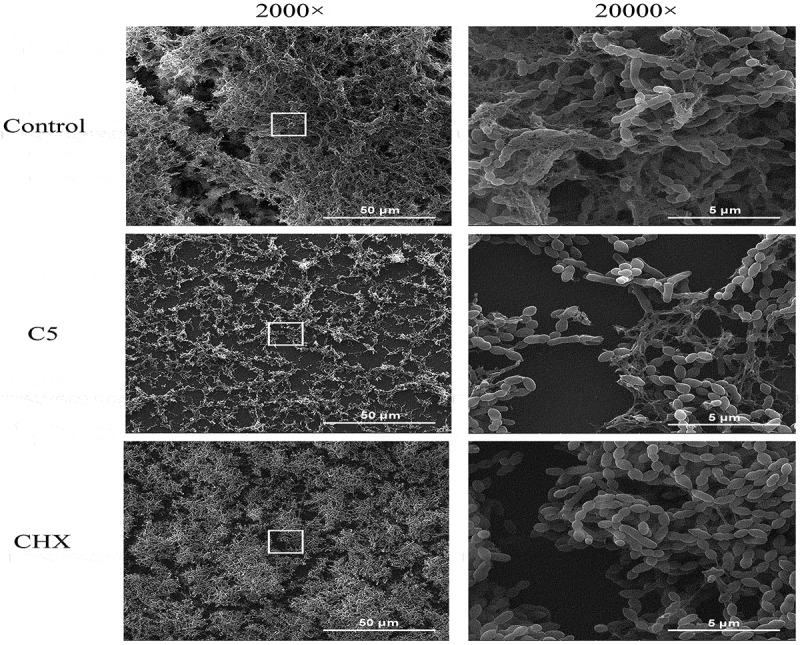
Morphological characteristics of multi-species biofilm in low and high magnifications images after the treatment of C5 and CHX. Microstructure was observed by SEM and white boxes in low magnification (left) were enlarged with a magnification of 20000 (right).

### Changes in the morphology of biofilm

C5 and CHX altered the morphology of multi-species biofilm captured by SEM (Figure 5). Clearly, in the picture at 2000x magnification, biofilm under C5 treatment resulted in the sparse and scattered microstructure. The thin-layer distribution was obvious, and the connections between cells were loose. In the presence of CHX, bacteria were in dense distribution, but bacterial cells were not encapsulated in the three-dimensional structure as control group. Meanwhile, in the picture at 20,000x magnification, although the morphology of the bacteria itself did not seem to be deformed in comparison with the control group, the extracellular polymer in C5 and CHX group was drastically reduced.

## Discussion

In the past decades, the development of novel complexes as antimicrobial agents has been extensively studied. The structure modification of imidazolium salts has been conducted. Although long alkyl chains contribute to the improvement of microbial inhibitory efficiency, they reduce the selectivity of mammals, which makes us no longer focus on long side chain imidazolium salts [31]. Therefore, we designed and synthesized several imidazolium salts with short side chains to evaluate their potential in plaque biofilm control. CHX, a cationic amphiphilic small-molecules, is used to prevent plaque accumulation and regarded as the gold-standard antiseptic [32,33]. Hence, it was selected as a comparative compound for the antimicrobial efficacy of the complexes we tested.

From the comparison of C1 and C2, it was shown that the acenaphthyl skeleton greatly enhanced the antimicrobial activity. In the presence of *ortho*-diisopropyl moieties, C3 exhibited efficient oral antimicrobial properties, especially against gram-positive *L. casei* and gram-negative *A.a*. Reasonably, it is presumed that *ortho*-diisopropyl moieties played an important role on acenaphthyl skeleton to enhance activities. In previous reports, several groups have revealed that the MIC required to inhibit the growth of microorganisms is related to the chain length of the N-substituted imidazolium salts, with the long alkyl chain (C8 to C16) imidazolium salts behaving the lowest MIC values [12,34]. Like quaternary ammonium compounds, imidazolium salts substituted with shorter alkyl chains have poorer surfactant properties and therefore lead to higher MIC values [35]. However, it was found that when the length of the carbon chain on N-moieties was increased further, the MIC did not decrease further. As illustrated in Table 2, the C4 with *iso*-pentyl on the N-aryl performed similar efficiency as C3.

Moreover, it is revealed that C4 and C5, derived from *ortho*-dialkyl with subtle modification, elicited excellent antimicrobial activity likewise. Greater antimicrobial activity was also observed compared with CHX against oral gram-positive pathogens. Expecting to obtain high-efficiency and low-toxicity small-molecule complexes, we further screened C3-C5 biofilm inhibitory effects on the major cariogenic *S. mutans* and their cytotoxic effects on HGECs. Intriguingly, C5 exhibited the same ability against *S. mutans* biofilm formation as CHX and lower cytotoxicity in the three imidazolium salts. Introduction of *Para*-methoxyl group facilitated the antimicrobial activity, which was already observed in other studies [36]. As electron donating groups, the attachment to the aromatic ring has an enhanced antimicrobial effect probably due to the high electron density causing inductive effect and better absorptivity [37]. According to our data, C5 was screened for further biofilm-related experiments.

During the process of biofilm formation to maturity, oral bacteria accumulate in an orderly manner, and the mutual adhesion between different bacteria contributes to the development of biofilm in space and time [21]. *S. gordonii* guided by a series of adhesin-receptor interactions and colonizes the tooth surface. As one of the initial oral colonization bacteria, it mediates the formation of early biofilm [38]. *A. naeslundii*, also one of the early colonizers on the tooth surface, can mediate the coaggregation of a variety of streptococci in the plaque biofilm [39]. Hence, we developed a multi-species biofilm consisting of *S. mutans, S. gordonii*, and *A. naeslundii*. Notably, C5 treatment could inhibit 50% biofilm at 0.98 μg/mL, while CHX with a 2-fold increased concentration.

In multi-species biofilm, *S. mutans* occupied an absolute dominance in the live bacteria without treatment. But over 90% of live *S. mutans* had been inhibited by the C5 and CHX treatment. This sharp decrease indicated these two agents could selectively kill *S. mutans* under selected concentrations, which may be due to the inhibit secretion of bioactive substances. Such as mutacin, secreted by *S. mutans*, is known to inhibit the growth of *S. gordonii* [40]. Furthermore, compared with *S. gordonii* in the control group, the number of which increased to a certain extent after C5 or CHX treatment. On the one hand, this proliferation may be due to the absence of *S. mutans* competing with nutritional substrates. Furthermore, as previously investigated by Jakubovics, coaggregation with *A. naeslundii* could enhance the growth and survival of *S. gordonii* [41]. In addition, C5 promoted the inhibition of *A. naeslundii*, which may represent the slow growth of *S. gordonii* than CHX. In general, the two compounds we evaluated showed the selectivity for *S. mutans* in multi-species biofilm, suggesting that they may not have deleterious effects on oral microbiota ecology.

The antimicrobial mode of CHX has been well clarified, as it leads to precipitation of the cytoplasmic contents, which results in the release of the main intracellular components and cell death [42,43]. Therefore, imidazolium salts, also as cationic complexes, may have a similar mode of CHX action. A recent study indicated that imidazolium salts in a relatively small molecular size can stimulate bacterial membranes thinning and enter the interior of the bacteria to interfere with the intracellular activities of the bacteria, exhibiting fast and efficient antimicrobial activity [44].

The extracellular polymer, particularly EPS synthesized by bacteria, contributes to bacterial colonization, biofilm formation and pathogenesis, and protects bacteria from desiccation, predation or antimicrobial agents [45,46]. Images observed by CLSM demonstrated that extracellular polymer in C5 and CHX group remarkably reduced, and it was likely the great reduction of *S. mutans*. Although fructans and soluble glucans synthesized by non-mutans streptococci and *A. naeslundii* may also contribute to the overall polysaccharide synthesis in the extracellular matrix, *S. mutans* is a key contributor to the formation of the EPS in dental biofilm [47]. The phenomenon of the reduction of extracellular polymer has been more intuitively reflected in the SEM images. Consistent with the qPCR results, SEM images confirmed the pronounced bacteria decrease in the presence of C5, the sparse structure also verified its anti-biofilm effectiveness . On the contrary, the stacked structure in the CHX biofilm may be due to the large amount of *S. gordonii*. However, given the microflora diversity of oral ecosystem, whether C5 treatment regulates microbial dysbiosis should be investigated by establishing a more intricate biofilm model. In addition, further studies focusing on underlying anti-biofilm mechanisms are still needed to be comprehensively explored.

Our research demonstrated the effectiveness of imidazolium salts against biofilm formation. Imidazolium salts are friendly for their flexible structural modifications compared with traditional antibiotics. Recently, a novel polymerizable imidazolium-containing resin with anti-biofilm properties and minimal cytotoxicity was developed, which may support imidazolium complexes as a platform for development of alternative antifouling biomaterials [48].

## Conclusion

In this study, imidazolium salts with varying skeleton and phenyl sidechain have been synthesized. It was observed that imidazolium salts with *ortho*-diisopropyl and *para*-methoxyl on N-moieties as well as bearing ancenaphthyl skeleton possessed excellent anti-biofilm efficiency and low cytotoxicity. These results indicate the high potential of the applicability of imidazolium salts as an oral antimicrobial agent.

## Supplementary Material

Supplemental Material
