# Supplementary material for: Anti-biofilm studies of synthetic imidazolium salts on dental biofilm in vitro
Source: J Oral Microbiol. 2022 May 17;14(1):2075309. doi: 10.1080/20002297.2022.2075309 (PMC9116249; doi:10.1080/20002297.2022.2075309)
Supplement: Supplemental Material [file ZJOM_A_2075309_SM8249.docx]

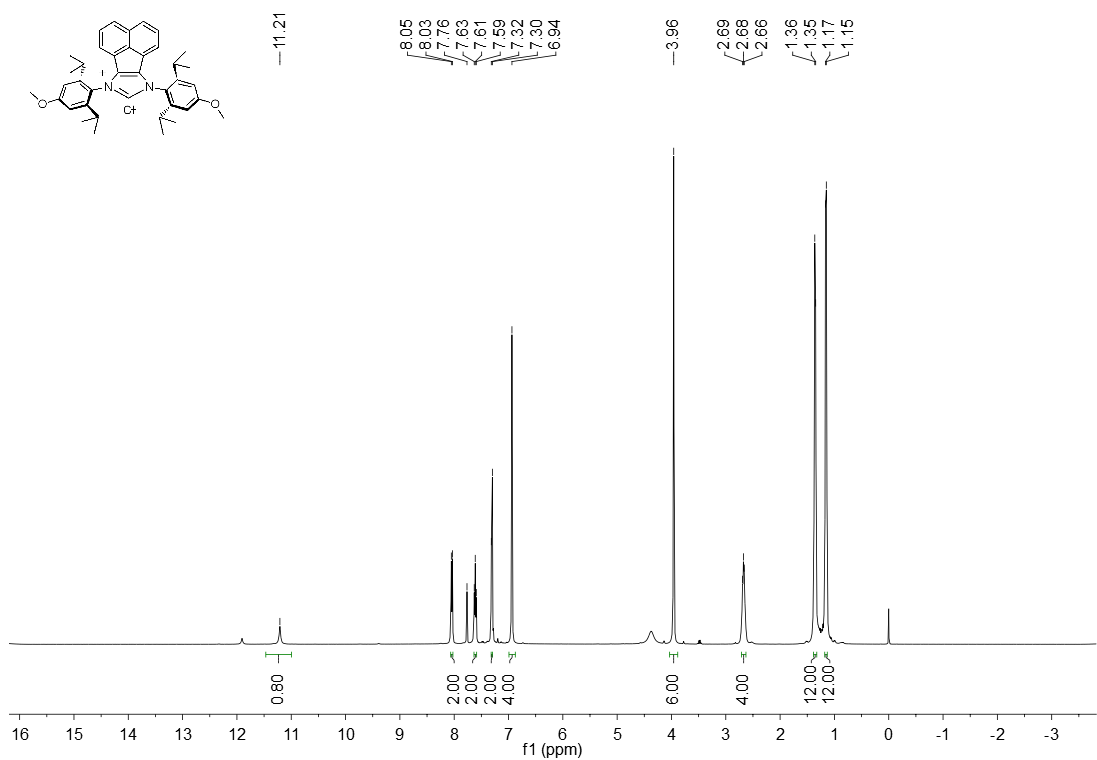


| **Figure S1.** The ^1^H NMR spectrum of C5.  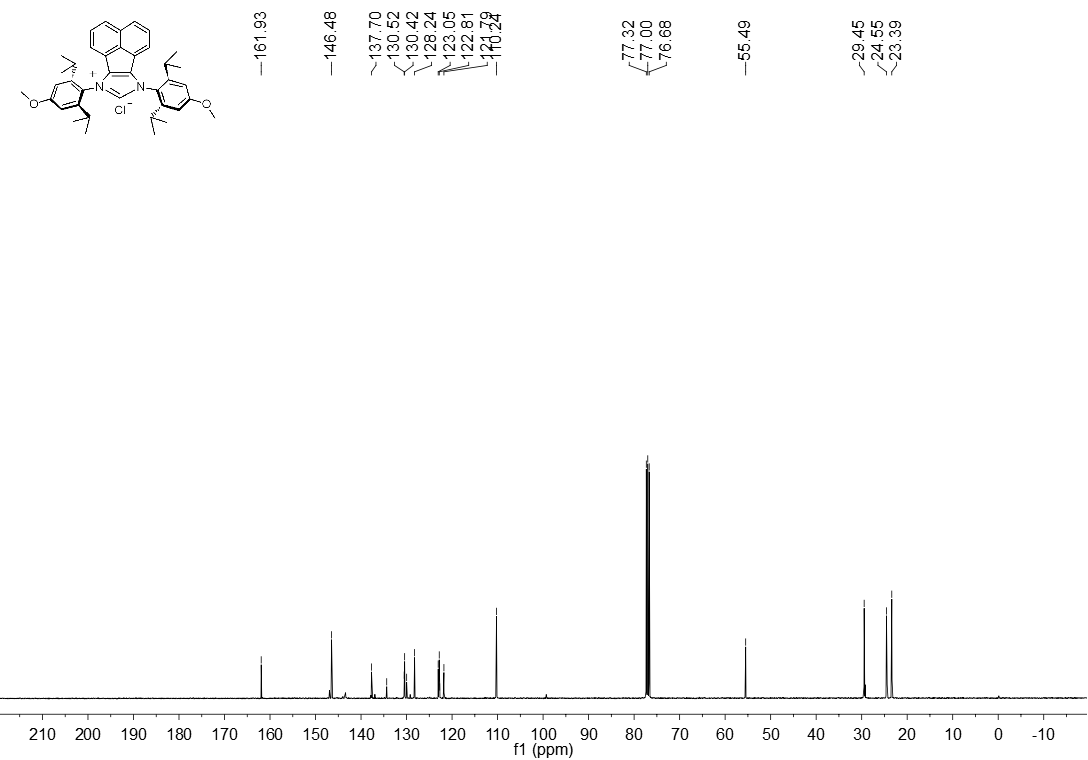  **Figure S2.** The ^13^C NMR spectrum of C5. |
| --- |

**Figure S3. standard curves of *S.gordonii*, *S.mutans* and *A.naeslundii***
